# ﻿*Argostemmaehuangzhangense* (Rubiaceae), a new species from Guangdong, China

**DOI:** 10.3897/phytokeys.214.89276

**Published:** 2022-11-30

**Authors:** Zhong-Cheng Liu, Jia Liu, Wan-Yi Zhao, Qiang Fan, Hua-Gu Ye, Lei Wang, Wen-Bo Liao

**Affiliations:** 1 State Key Laboratory of Biocontrol and Guangdong Provincial Key Laboratory of Plant Resources, School of Life Sciences, Sun Yat-Sen University, Guangzhou 510275, China Capital Normal University Beijing China; 2 College of Resource Environment and Tourism, Capital Normal University, Beijing 100048, China Sun Yan-Sen University Guangzhou China; 3 Key Laboratory of Plant Resources Conservation and Sustainable Utilization, South China Botanical Garden, Chinese Academy of Sciences, Guangzhou 510650, China South China Botanical Garden, Chinese Academy of Sciences Guangzhou China

**Keywords:** *Argostemmaehuangzhangense*, China, Guangdong, new species, Rubiaceae

## Abstract

*Argostemmaehuangzhangense*, a new Rubiaceae species from E’huangzhang Nature Reserve, Guangdong Province, China, is here described and illustrated. A morphological comparison between the new species and its putative relatives, *A.lamxayanum*, *A.laotica* and *A.verticillatum*, is presented. The new species is mostly similar to *A.laotica*, but they can be distinguished from each other since *Argostemmaehuangzhangense* presents solitary flower (vs. 2-flowered inflorescences), flower lobes 4 (vs. 5) and anthers opening by longitudinal slits (vs. apical pores). In a preliminary IUCN Red List status of *Argostemmaehuangzhangense* this species is assigned as Vulnerable (VU).

## ﻿Introduction

The genus *Argostemma* Wall. (Wallich 1824) belongs to the coffee family, Rubiaceae (subfamily: Rubioideae), and in its own tribe Argostemmateae (Bremer 1987; Bremer and Manen 2000). *Argostemma* is a large genus of more than 160 species and is widely distributed in the Old World tropics with most species occurring in SE Asia and two species in west tropical Africa (Bremer 1989; Sridith and Puff 2000; Mabberley 2017; Lanorsavanh et al. 2020). In China, six species of *Argostemma* were recorded (Chen and Taylor 2011). Key morphological characters of *Argostemma* are (i) opposite or verticillate leaves that are slightly to markedly anisophyllous, (ii) 4- or 5-merous flowers without nectaries, (iii) white and rotate corollas, (iv) Inner surface of corolla tube glabrous, (v) free anthers or coherent into a tube, (vi) anthers with opening by longitudinal slits or apical pore, and (vii) sometimes connective prolonged at the apex (Puff et al. 1995; Chen and Taylor 2011).

An unknown *Argostemma* species was discovered during recent field surveys conducted between April 2017 and May 2018 at the E’huangzhang Nature Reserve, Yangchun City, Guangdong Province. The flowers of the unknown *Argostemma* species are clearly 4-merous and it differs from all known *Argostemma* species in China. In addition, we compared the unknown *Argostemma* from E’huangzhang Nature Reserve against *Argostemma* species occurring in Southeastern Asian which presented morphological characters divergent from those in our specimen. Thus, it was concluded that the unknown *Argostemma* from E’huangzhang Nature Reserve is a new species, which is hereby described and illustrated here.

## ﻿Materials and methods

This study was conducted based on living and dried herbarium specimens collected from nearby Wufu Waterfall in the E’huangzhang Nature Reserve. Herbarium specimens at IBSC, KEP and KUN, as well as types at IBSC, K and NY, were examined for morphological comparisons (acronyms follow Thiers 2022). Taxonomic literature of the genus for Thailand (Sridith and Puff 2000; Sridith and Larsen 2004; Sridith 2007, 2009, 2012), China (Chen and Taylor 2011), Vietnam (Vu et al. 2020), Laos (Lanorsavanh and Chantaranothai 2013, 2016, 2019; Lanorsavanh et al. 2020), and Myanmar (Kress et al. 2003; Tanaka et al. 2010) were consulted.

## ﻿Results

*Argostemmaehuangzhangense* is similar to *A.lamxayanum* and *A.laotica* by sharing the following morphological features: leaves isophyllous, verticillate or pseudo-verticillate; and leaves, pedicels, calyx and petals pubescent (Table 1). However, *Argostemmaehuangzhangense* is distinct from these species by its flower solitary (vs. flower (1-)2–10); peduncles absent (vs. peduncles short or 1–3.5 cm); flower 4-merous (vs. 5-merous); anther length 1.2–1.5 mm (vs. longer than 2 mm) and anthers longitudinal slits (vs. apical pore) (Table 1).

**Table 1.** Comparison of morphological characters between *Argostemmaehuangzhangense*, *A.lamxayanum*, *A.laotica* and *A.verticillatum*.

Characters	*A.ehuangzhangense*	*A.lamxayanum*	*A.laotica*	*A.verticillatum*
Plant height (cm)	1–4	2–12	1–2.5	2–10
Leaf blade shape	ovate to elliptic	elliptic, oblong or ovate	elliptic or oblanceolate	lanceolate or ovate-lanceolate
Leaf blade size (cm)	0.5–2.5×0.3–1.2	0.7–4×0.4–2.0	1–1.7×0.4–0.7	1–7× 0.7–2.5
Lateral leaf veins	3–4-paired	4–8-paired	3–4-paired	4–7-paired
Leaf indumentum	both surfaces antrorse strigose (abaxial sparsely pubescent on the vein)	both surfaces hirsute	both surfaces pubescent	both surfaces glabrous or sparsely pubescent
Inflorescences	flowers solitary; peduncles absent	umbelliform,1–10-flowered; peduncles 1–3.5 cm, glabrous	flowered; peduncles very short, pubescent	umbelliform, 1–3-flowered, composed cymose; peduncles 1–3 cm long, glabrous
Pedicel	8–18 mm, pubescent	4–12 mm, pubescent	6–7 mm, pubescent	5–10 mm, glabrous
Calyx lobes	4, pubescent outside	5, pubescent outside	5, pubescent outside	5, glabrous both side
Corolla lobes	4, pubescent outside	5, pubescent outside	5, pubescent both side	5, glabrous both side
Filament length (mm)	3.5–4	2.5–3	2.5–3.2	1–1.2 (-2.5)
Anthers coherence	connivent	free	connivent	free
Anther length (mm)	1.2–1.5	2.5–3	2.2–2.5 mm	2–3
Anther dehiscence	longitudinal slits	apical pores	apical pores	apical pores

Amongst the *Argostemma* species known in China, *A.verticillatum* is morphologically similar to *Argostemmaehuangzhangense*. *Argostemmaverticillatum* differs from the latter by its (i) glabrous stem, pedicels and calyx, (ii) inflorescence cymose and comprised of 1–3 umbelliform, (iii) flowers 5-merous, and (iv) filaments short (Table 1).

### ﻿Taxonomic treatment

#### ﻿ Argostemma ehuangzhangense

Taxon classificationPlantaeGentianalesRubiaceae

H.G.Ye, Jia Liu & W.B.Liao sp. nov.

C638A75A-D715-56E9-8B26-3875DC51DC3D

urn:lsid:ipni.org:names:77309064-1

##### Type.

**Figure 1.**
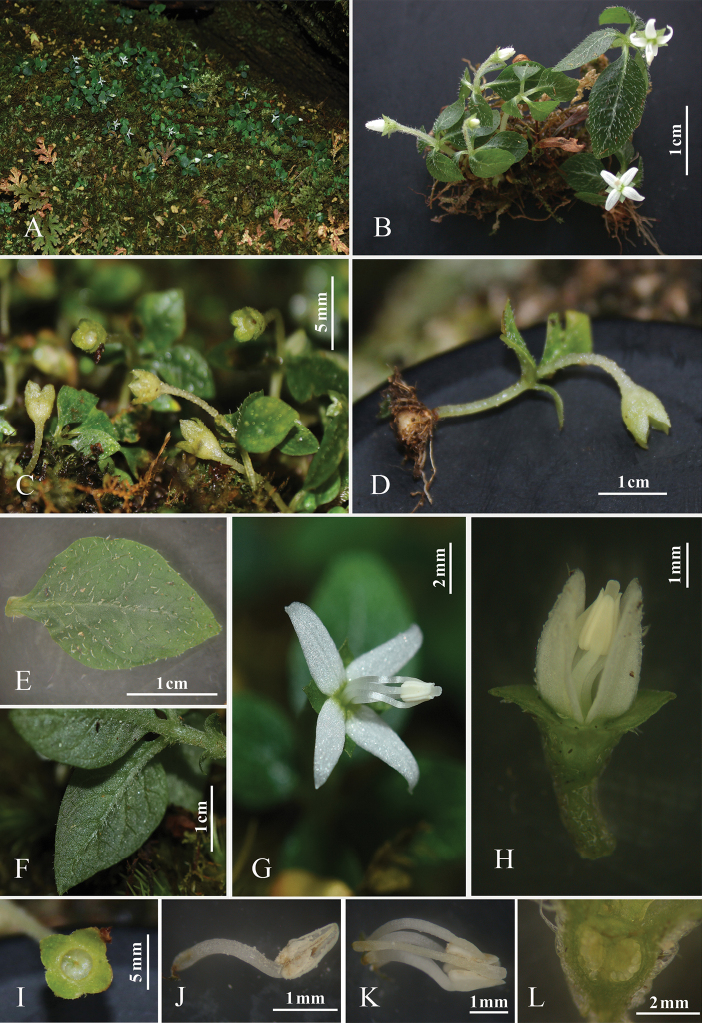
*Argostemmaehuangzhangense***A** individuals in their natural habitat **B** flowering individuals **C** fruiting individuals **D** side view of an individuals with tuber **E** leaf blade adaxial surface view **F** leaf blade abaxial surface view **G** flower, internal corolla surface view **H** folwer, external corolla surface view **I** capsule, top view **J** stamen **K** style and stigma tightly enclosed by stamens **L** ovary longitudinal section view. (photographs (**A–G**) were taken by Wan-Yi Zhao in the original habitat area of E’huangzhang and photographs (**H–L**) were taken by Jia Liu in SYS Herbarium in May 2018).

**Figure 2.**
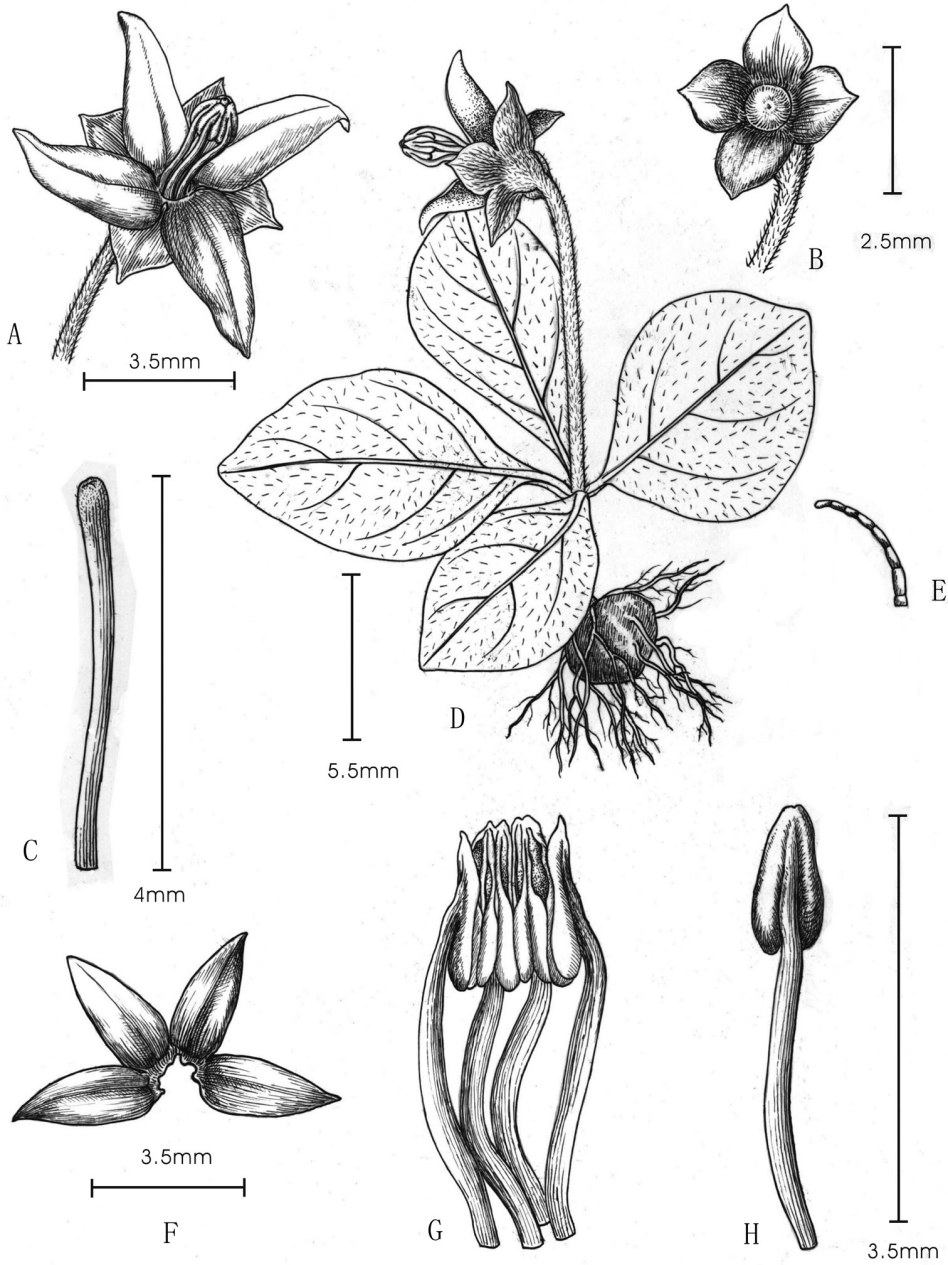
*Argostemmaehuangzhangense***A** flower **B** fruit **C** style and stigma **D** habit of a mature plant **E** multicellular trichomes present on the stem, pedicel and ovary **F** corolla **G** organization of stamens **H** stamen (Drawn from living plants by Yun-Xiao Liu).

China. Guangdong Province: Yangchun City, Bajia Town, E’huangzhang Nature Reserve, near the Wufu Waterfall, 21°52'N, 111°25'E, a.s.l. 720 m, 3 May 2018, *Wan-Yi Zhao, Jia Liu, Qiao-Ling Ding, Fan Ye YC-2018-02* (holotype: SYS!, Barcode SYS00236851; isotype: SYS!, Barcode SYS00236852) (Figs 1, 2).

##### Diagnosis.

*Argostemmaehuangzhangense* is similar to *A.lamxayanum* and *A.laotica* in its habit and pseudo-verticillate leaves, but differs in having terminal solitary 4-merous flowers, short anthers (1.2–1.5 mm long) opening by longitudinal slits.

##### Description.

***Terrestrial*** perennial herbs, 1–4 cm tall, attached to the substrate by tubers flattened globose, with a few roots. ***Stems*** erect, densely pubescent, with one pair of scale-like leaves at the lower middle portion. ***Leaves*** four per individual, clustered at the stem apex, verticillate, anisophyllous, petiole 0.5–2 long mm; blades membranous or thinly papery when dried, ovate to elliptic, 5–25 × 3–12 mm, cuneate at base, acute to obtuse at apex, margins entire; adaxial surface green, densely antrorse strigose; abaxial surface grey-white with white particles, sparsely pubescent on the midrib vein and lateral veins; lateral veins 3- or 4-pairs; stipules deciduous. ***Flowers*** solitary, terminal; pedicels 0.8–1.8 cm, with densely pubescent, trichomes multicellular. ***Calyx*** densely pubescent, trichomes multicellular, hypanthium portion obovoid; lobes 4, subtriangular, 1–1.3 × 1.1–1.4 mm, abaxially pubescent, adaxially glabrous. ***Corolla*** white, rotate, external surface sparsely pubescent, internal surface glabrous, corolla tube 0.3–0.6 mm long; corolla lobes 4, oblong-lanceolate, 3–4 × 1.5–2 mm. ***Stamens*** 4; filaments free, 3.5–4 mm, exserted; anthers 1.2–1.5 mm long, coherent into a tube, dehiscent longitudinally. ***Ovary*** 2-locular, ovules numerous in each locule; style filiform, ca. 4 mm, with short pubescence, stigma capitate, exserted. ***Capsule*** obovoid, 2–5 mm in diameter, 1–3 mm long, pubescent, crowned by a persistent calyx, without ribs or furrows.

##### Phenology.

This species is recorded flowering in March-May and fruiting in May-September.

##### Distribution.

*Argostemmaehuangzhangense* is endemic to E’huangzhang Nature Reserve, southwestern Guangdong Province. It is currently known only from two populations recorded in county of Dianbai and city of Yangchun.

##### Habitat.

Growing along river on wet sandstone under the evergreen broad-leaf forest at 400–750 m a.s.l.

##### Etymology.

The specific epithet ‘ehuangzhangense’ is derived from the type locality, E’huangzhang Nature Reserve of the Guangdong Province, in China. This area is the oldest geological platform in the Guangdong Province, in which many endemic species occur (Wang et al. 2003; 2004; Ding et al. 2018). The new species is also expected to occur in the Yunkaishan National Nature Reserve, Maoming City, because this area shares a similar tectonic history with E’huangzhang. Therefore, we proposed for the vernacular name of the species as yuèxīxuěhuā (粤西雪花).

##### Preliminary conservation status.

The species is endemic to the Guangdong Province. According to our field survey, there are only two localities in which *Argostemmaehuangzhangense* is recorded and each population consists of 100–250 individuals. The number of mature individuals of *Argostemmaehuangzhangense* is more than 400, but less than 1000. Thus, we believe that *Argostemmaehuangzhangense* would be considered VU (Vulnerable) in an official IUCN Red List assessment (IUCN Standards and Petitions Subcommittee 2022) according to the D criterion.

##### Paratypes.

China. Guangdong Province: Yangchun City, Bajia Town, E’huangzhang Nature Reserve, 21°52'N, 111°25'E, a.s.l. 704 m, 29 Apr. 2017, *Hua-Gu Ye*, *Zhong-Cheng Liu YHG-06* (SYS); same locality, 5 Aug. 2017, *Hua-Gu Ye*, *Wan-Yi Zhao*, *Zhong-Cheng Liu YC2017-35* (SYS); Yangchun City, Bajia Town, 21°52'N, 111°25'E, a.s.l. 750 m, 1 Aug. 2001, *Hua-Gu Ye 6119* (IBSC); Dianbai County, Luokeng Town, Shuangjifeng, 21°52'N, 111°21'E, a.s.l. 400 m, 8 Aug. 2001, *Hua-Gu Ye 6427* (IBSC).

## Supplementary Material

XML Treatment for Argostemma ehuangzhangense
